# Balb/c FasKO mice develop allergic blepharitis associated with hyper-production of IgE

**DOI:** 10.1186/ar3620

**Published:** 2012-02-09

**Authors:** Ayumi Fukuoka, Shizue Yumikura-Futatsugi, Suzuka Takahashi, Hirotaka Kazama, Kenji Nakanishi, Shin Yonehara

**Affiliations:** 1 Graduate School of Biostudies, Kyoto University, Japan; 2 Department of Immunology and Medical Zoology, Hyogo College of Medicine, Japan; 3 Institute of Genome Reserch, The University of Tokushima, Japan

## 

Fas is a member of the TNF receptor family and crucial for induction of apoptosis. MRL- *lpr*/*lpr *mice, which carry a mutation of Fas, spontaneously develop systemic autoimmune disease including arthropathy, indicating that Fas plays an important role in elimination of self-reactive immunocytes by apoptosis. In addition to autoimmune diseases, we found a novel phenotype of FasKO mice exclusively in Balb/c genetic background that is allergic blepharitis. Allergic blepharitis is revealed in Balb/c FasKO mice from 15 week-old and about 85% of the mice suffered from allergic blepharitis at 35 week-old. Serum concentrations of both IgG1 and IgE Abs were about 100-times higher in 20-week old FasKO mice than in WT mice; however, there was no significant difference between WT and FasKO mice in the ability of B cells to produce IgG1 and IgE Abs in the presence of IL-4 and anti-CD40 Ab inducing co-stimulatory signals. Additionally, the production of IL-4 by T cells was same. These results suggested that other type of cells enhanced IgG1 and IgE Abs production from B cells in Balb/c FasKO mice. To identify the cells enhancing IgG1 and IgE Abs production, we cultured B cells *in vitro *in the presence of IL-4 and anti-CD40 Ab together with various types of cells from Balb/c FasKO mice. In the result, we found FasKO non-T non-B cells upregulated the production of both IgG1 and IgE from B cells. Moreover, the number of these cells was specifically increased in Balb/c FasKO mice.All the results indicate that these cells enhance production of IgG1 and IgE from B cells in the presence of IL-4 and anti-CD40 Ab, and excessive accumulation of these cells may cause allergy *via *hyper-production of IgE.

**Figure 1:**
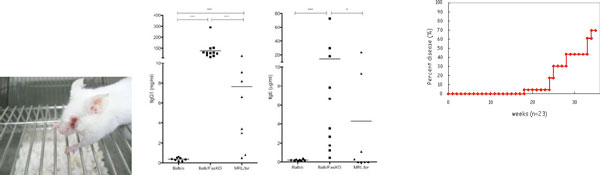
**Balb/c FasKO mice develope allergic blepharitis**.
